# Identification of novel candidate loci for Alzheimer’s disease and related dementias by leveraging the shared genetic basis with hippocampal volume

**DOI:** 10.1016/j.nbas.2025.100147

**Published:** 2025-07-30

**Authors:** Chenyang Jiang, Sven J. van der Lee, Niccolò Tesi, Wiesje M. van der Flier, Betty M. Tijms, Lianne M. Reus

**Affiliations:** a Alzheimer Center Amsterdam, Neurology, Vrije Universiteit Amsterdam, Amsterdam UMC location VUmc, Amsterdam, the Netherlands; b Amsterdam Neuroscience, Neurodegeneration, Amsterdam, the Netherlands; c Section Genomics of Neurodegenerative Diseases and Aging, Department of Clinical Genetics, Vrije Universiteit Amsterdam, Amsterdam UMC, De Boelelaan 1117, 1081HV Amsterdam, the Netherlands; d Delft Bioinformatics Lab, Delft University of Technology, Delft, the Netherlands

**Keywords:** Hippocampus, Alzheimer’ disease, Genetic risk factors

## Abstract

Alzheimer’s disease and related dementias (ADRD) are complex neurodegenerative disorders of which the genetic basis remains incompletely understood. Hippocampal volume loss is a core hallmark of AD. Hippocampal volume also has a strong heritable component and its genetic underpinnings may help us to understand the complex biological mechanism underlying ADRD. To identify shared genetic risk loci across late-onset ADRD and bilateral hippocampal volumes, we conducted a cross-trait analysis of existing GWAS data on the two traits using the conjunctional false discovery rate (conjFDR) framework. Functional annotation and phenome-wide association studies (PheWAS) were performed on the identified shared loci to characterize their biological relevance. We identified 11 unique lead genetic loci, of which 7 loci showed discordant directional effects (loci associated with increased risk for ADRD and smaller hippocampal volumes). We found that *SHARPIN* and *TNIP1* genes play a role in ADRD by affecting hippocampal volumes. In addition, we observed 9 novel ADRD-hippocampus loci in genes previously implicated in AD (*IGIP* and *ACE*) and novel ADRD-genes (*KCTD13*, *HINT1*, *SH3TC2*, *FAM53B*, *TPM1*, *IL34* and *SSH2*). PheWAS results show that most shared loci associated with neuroimaging measurements, white blood cell markers, red blood cell markers, and lipids. This study shows a shared genetic basis between ADRD and bilateral hippocampal volumes. By integrating summary statistics for these two traits, we identified both novel and previously reported ADRD-hippocampus loci. Functional analysis highlights the role of immune cells and lipid markers in the shared loci, suggesting a shared neurobiological basis for ADRD and bilateral hippocampal volumes.

## 1 Introduction

Alzheimer’s disease and related dementias (ADRD) are progressive and irreversible brain disorders affecting over 50 million people worldwide [1]. Genome-wide association studies (GWAS) have identified over 80 genetic variants associated with ADRD, but together these explain only a small fraction of its high heritability of ∼ 58–78 % [2]. The identification of more ADRD risk loci will increase our understanding of the underlying disease mechanisms, which is important for the development of treatment targets and strategies.

A core symptom of Alzheimer's disease (AD) is memory impairment, which is closely related to hippocampal atrophy. Hippocampal volumes can be assessed using structural magnetic resonance imaging (MRI) [3,4], and previous studies have indicated that this measure is highly heritable, with heritability estimates ranging from 60 %-85 % in twin studies, and 17 % based on SNP-based heritability [5,6]. Prior studies in young adults suggested that the genetic markers for AD confer life-long susceptibility to different brain structure: both major genetic risk factor for AD Apolipoprotein E (*APOE*)-e4, and *APOE*-excluded polygenic risk score (PRS) have independent effects on hippocampal volumes [[7], [8], [9], [10]]. These observations suggest that the genetic architecture of AD overlaps with genetic variation that influence individual variability in hippocampal volumes.

One way to study shared genetic architecture between two traits is through conjunctional false discovery rate (conjFDR) analysis, which aims to enhance the identification of shared individual genetic variants by leveraging the collective power of two GWAS studies [11]. Previous studies using this approach in psychiatric disorders indicated that this could leverage auxiliary genetic information and brain morphology [12,13], leading to the identification of novel loci [14]. We hypothesized that combining genetic data of ADRD and hippocampal volume, one of the main affected brain signatures in ADRD, could improve the detection of novel AD-associated genetic markers.

In this study, we examined this hypothesis on the identification of shared genetic risk loci, by conducting a conjFDR analysis on the most recent GWAS on ADRD and bilateral hippocampal volumes [6,15]. To identify candidate genes with functional relevance, we then performed the downstream analyses of the discovered loci in Genotype-Tissue Expression (GTEx) data and other databases. The results of this study may elucidate pleiotropic effects on ADRD and hippocampal volumes that offer insights into causal neuropathological pathways underlying both traits.

## 2 Materials and methods

### 2.1 GWAS summary statistics

GWAS summary statistics were obtained from GWAS catalog [https://www.ebi.ac.uk/gwas/] for the ADRD GWAS[15] and Oxford Brain Imaging Genetics Server [https://open.win.ox.ac.uk/ukbiobank/big40/] for the GWAS on bilateral hippocampal volumes [6]. Descriptive statistics for the GWASs used in this study are provided in Supplementary Table S1. Details for the genotyping procedure, quality control, and GWAS analysis are provided in the primary manuscripts for the ADRD GWAS and the hippocampal volumes GWAS. The ADRD GWAS summary statistics included association P values, beta coefficients, standard errors, and effect alleles. This study included 85,934 clinically diagnosed/proxy AD cases and 401,577 controls, and found 75 AD-related risk loci at stage 1 analysis. Within ADRD GWAS analysis, individuals met standard clinical criteria for AD—most commonly the NINCDS-ADRDA criteria for “probable” or “definite” AD (or equivalent diagnostic frameworks such as DSM-IV and CERAD)—often supplemented by neuroimaging and, in some studies, neuropathological confirmation, and in UK Biobank, “proxy” cases were defined by self-reported parental history of Alzheimer’s disease or dementia (i.e. participants whose mother or father had dementia) [6,16]. Both GWAS studies are based on European ancestry cohorts. The left and right hippocampal volumes GWAS summary statistics included association P values, beta coefficients, standard errors, and effect alleles based on 39,691 participants of the UK Biobank. The original UKB data includes six MRI modalities: T1-weighted and T2-weighted fluid-attenuated inversion recovery (T2-FLAIR) structural images, susceptibility-weighted MRI, diffusion MRI, task functional MRI and resting-state functional MRI. The detailed processing could be found in UKB brain imaging documentation (https://biobank.ctsu.ox.ac.uk/showcase/showcase/docs/brain_mri.pdf).

Quality control (included sanity check and duplication removing) of the GWAS summary statistics was conducted in the publicly available toolbox GWASlab [17] [https://github.com/Cloufield/gwaslab]. Coordinates of SNPs in the ADRD GWAS were lifted from hg38 to hg19 to align with the default genome assembly of the conjFDR software. We removed four regions with complex Linkage Disequilibrium (LD) patterns, including major histocompatibility complex region: 6:25119106–33854733; 8p23.1: 8:7200000–12500000; *MAPT* region: 17:40000000–47000000; and the *APOE* region: 19:45111942 – 45711941. A total of 12,025,887 SNPs were common in the two GWAS that were used for further analysis.

### 2.2 Shared locus discovery

We applied conjFDR analysis to explore the shared genetic variants across the ADRD and bilateral hippocampal volumes GWAS [11]. The conjFDR analysis is built on an empirical Bayesian statistical framework and it leverages cross-trait SNP enrichment to improve the power to discover variants with a shared effect. We first used SNP associations of the hippocampal volumetric phenotypes to re-rank test statistics and re-calculate the significance of associations between these SNPs and ADRD. Then, we inverted the roles of two phenotypes to re-calculate the significance of SNP associations to the brain volumetric phenotypes. The conjFDR analysis conservatively estimates the posterior probability that a SNP has no association with either trait, represented as a conjFDR value. This method can effectively boost the GWAS discovery power by leveraging auxiliary genetic information contained in a highly phenotypic-related trait. SNPs with a significance of conjFDR < 0.05 and having LD r2 < 0.1 within 1000 kb were defined as independent significant signals with ‘–clump’ function in PLINK[18].

### 2.3 Co-localization analysis

To examine whether shared loci identified in the conjFDR analysis could be causal to both ADRD and bilateral hippocampal volumes, we performed colocalization analysis using coloc[19] for all shared loci. Briefly, coloc uses a Bayesian approach to estimate the posterior probability (PP) that a given genetic locus is not associated with ADRD or bilateral hippocampal volumes (hypothesis 0 [H0]), is uniquely associated with ADRD (H1) or bilateral hippocampal volumes (H2), harbors two independent causal variants for each phenotype (H3), or harbors a shared causal variant for both ADRD and bilateral hippocampal volumes (H4). Coloc anlaysis evaluated evidence of colocalization within a 500 kb region centered at each shared risk locus. The posterior probabilities for H4, i.e., there is a similar causal locus for both ADRD and bilateral hippocampal volumes, were used to evaluate the evidence for colocalization of shared loci.

### 2.4 Functional interpretation of candidate shared genetic variants

To study the potential functional consequences of loci shared across ADRD and bilateral hippocampal volumes, we used the functional annotation section of the snpXplorer[20]web server with default settings. This tool performs gene annotation through two approaches: (1) assigning the nearest gene to each variant, and (2) mapping variants to genes based on functional consequences, incorporating CADD scores[21], and quantitative-trait-loci analyses (expression-QTLs and splicing-QTLs). Given the large number of variants associated with gene expression, we further performed colocalization analyses between shared loci and eQTL signals from GTEx v8 database[22] to identify the possible candidate genes. To further characterize the significant shared loci, we conducted phenome-wide association studies (PheWAS). Briefly, PheGWAS is a powerful tool for exploring the associations between genetic markers and a wide range of traits, and can provide insights into the complex relationships between genetics and phenotypic traits. The traits that are associated with the list of SNPs with P < 1 × 10 –5 in all GWAS harmonized summary statistics in the MRC IEU OpenGWAS data infrastructure were extracted by using ‘phewas’ function of the R-package ‘ieugwasr’[23]. We performed quality control on the PheWAS results, which included standardizing trait names, filtering out traits with a sample size of less than 50,000 due to their low statistical power, and removing duplicated SNP-trait associations. To identify key pleiotropy, we selected the top three traits for each shared locus based on P-values for visualization.

We then assessed whether our lead variants are also associated with AD–related endophenotypes by leveraging two complementary resources. First, we examined cerebrospinal fluid (CSF) biomarkers—amyloid-β, total tau, and phosphorylated tau (pTau)—using summary statistics from an in-house GWAS of 18,948 samples [24]. Second, we evaluated AD related neuropathology including CERAD scores for neuritic plaques, quantitative measures of amyloid-β deposition, and Braak neurofibrillary tangle (NFT) stage—based on the data of Shade et al. [25]. For each lead variant, we extracted P values and effect size estimates from both datasets, then compared the effect sizes and direction of association. We considered associations to be suggestive at P < 0.10 for both the CSF endophenotype GWAS and the neuropathology analyses.

### 2.5 Patient consent

Participants who contributed to cohort-level summary statistics constituting the *meta*-analyses provided written informed consent. All contributing cohorts were approved by their local institutional review board.

## 3 Results

### 3.1 conjFDR analysis identifies 11 genetic loci shared across ADRD and bilateral hippocampal volumes

**Fig. 1:**
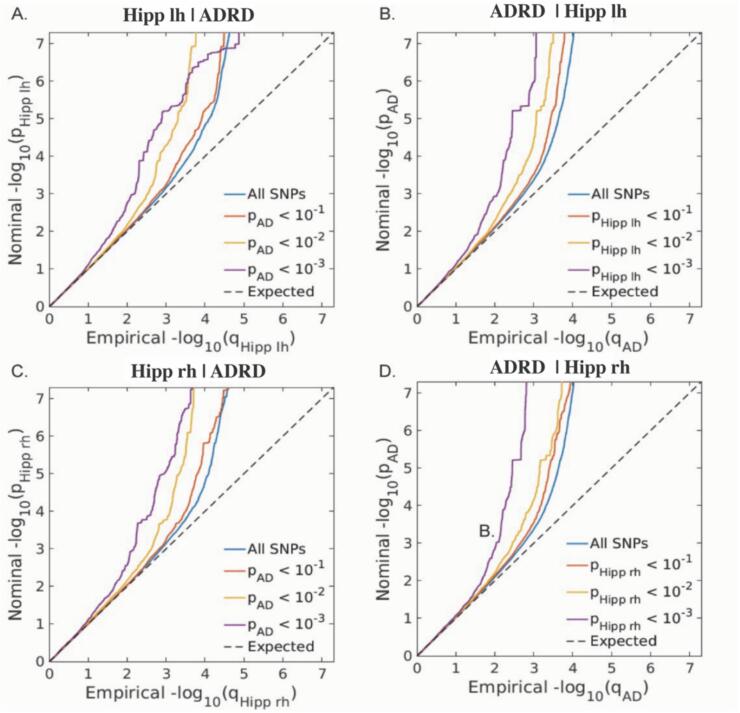
Conditional quantile–quantile plots. Conditional Q–Q plots of nominal versus empirical ADRD − log10p values below the standard GWAS threshold of P < 5 × 10^−8^ as a function of significance of association with bilateral hippocampal volumes, at the level of P ≤ 0.1, P ≤ 0.01, P ≤ 0.001, respectively (left panel). Conditional Q–Q plots of nominal versus empirical bilateral hippocampal volumes − log10p values below the standard GWAS threshold of P < 5 × 10^−8^ as a function of significance of association with ADRD, at the level of P ≤ 0.1, P ≤ 0.01, P ≤ 0.001, respectively (right panel). The dashed lines indicate the null hypothesis. The blue line shows association P values in the main trait of interest (ADRD) for all SNPs irrespective of their association P values in hippocampal volumes. Red, yellow, and purple lines show association P values in the main trait for successively nested subsets of SNPs with increasing significance in the conditional trait. The consistently increasing degree of leftward deflection for subsets of variants with higher significance in the conditional trait in both directions indicates substantial polygenic overlap between ADRD and hippocampal volumes.

**Fig. 2:**
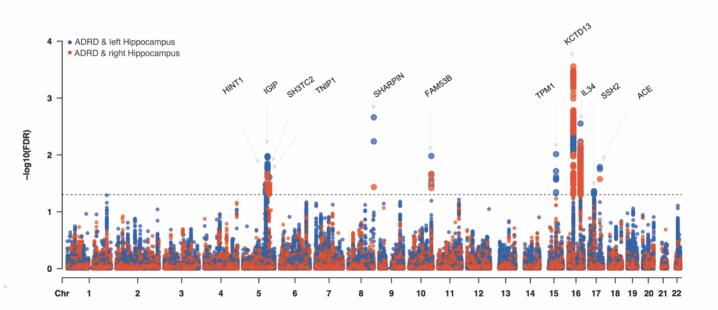
Shared genetic variants jointly associated with Alzheimer’s disease and related dementias and bilateral hippocampal volumes. The x-axis stands for the base pair position and y-axis represents −log10 transformed conjunctional false discovery rate values with a black horizontal line reflecting significance. Lead signals within LD blocks located less than 250 kb apart and associated with both left and right hippocampal volumes are merged into a single locus. Abbreviations: KB: Kilobyte; Chr: chromosome; FDR: false discovery rate; ADRD: Alzheimer's disease and related dementias; conjFDR: conjunctional false discovery rate; LD: linkage disequilibrium.

We applied conjFDR analysis to identify shared genetic variants across ADRD and bilateral hippocampal volumes. To provide a visual pattern of pleiotropic enrichment between the two phenotypes, we generated conditional quantile–quantile (Q–Q) plots (Fig. 1) from the conjFDR results. The conditional Q–Q plots showed a strong enrichment pattern for ADRD given hippocampal volumes, and vice versa. SNPs with a significance of conjFDR < 0.05 and LD r2 < 0.1 within 1000 kb were defined as independent significant signals. We identified 10 lead loci shared by ADRD and left hippocampal volume, and 8 common loci shared by ADRD and right hippocampal volume (Supplementary Table S2). After merging lead signals for left and right hippocampal volumes with similar LD patterns (i.e., LD blocks closer than 250 kb), we identified 11 unique lead loci shared by ADRD and bilateral hippocampal volumes (see Table 1 for a list of all shared loci). We then compared the effect direction on ADRD risk and hippocampal volumes. There were 7 loci associated with increased risk for ADRD and smaller hippocampal volumes, whereas 4 loci associated with increased ADRD risk and larger hippocampal volumes (Fig. 2).

**Table 1:** Common lead loci shared with Alzheimer’s disease and related dementias and bilateral hippocampal volumes.

**CHR**	**BP**	**Variant**	**Effect/Other allele**	**Consequence**	**Gene**	**Directions ADRD/Hippocampal volume**	**conjFDR**	**Hippocampus**
5	130096260	rs12518350	A/G	intergenic	*HINT1*	-/+	0.0329	lh
5	139518989	rs156092	T/C	intergenic	*IGIP*	-/-	0.0215	lh/rh
5	148430451	rs36080	A/G	intron	*SH3TC2*	-/+	0.0351	rh
5	150432388	rs871269	T/C	regulatory	*TNIP1*	-/+	0.0245	lh/rh
8	145158607	rs34173062	A/G	regulatory	*SHARPIN*	+/-	0.00216	lh/rh
10	126358314	rs61870529	T/C	intron	*FAM53B*	+/-	0.0105	lh/rh
15	63379726	rs12904537	T/G	regulatory	*TPM1*	+/-	0.00977	lh
16	29943367	rs12919683	T/C	downstream	*KCTD13*	+/+	0.00028	lh/rh
16	70671422	rs139473334	A/AT	regulatory	*IL34*	-/-	0.00283	lh/rh
17	61575854	rs149155892	T/C	regulatory	*ACE*	+/-	0.0162	lh/rh
17	28113123	rs2628166	T/C	intron	*SSH2*	+/+	0.0439	lh

Mapped gene: positional nearest gene; Directions ADRD/Hipp_vol: The directions of Z score in original ADRD and bilateral hippocampal volume GWAS. The colored rows show concordant effects in ADRD and bilateral hippocampal volume, indicating that increased/decreased risk for ADRD associates with smaller/larger hippocampus volumes. ADRD: Alzheimer’s disease and related dementias, CHR: chromosome, BP: base pair position, conjFDR: conjunctional false discovery rate, lh: left hemisphere, rh: right hemisphere.

### 3.2 Shared ADRD-hippocampus loci act as quantitative trait loci (QTLs)

To gain more insight into the potential functional consequences of the 11 lead SNPs for ADRD and bilateral hippocampal volumes, we performed positional, eQTL, and sQTL mapping using existing gene expression databases[22,20]. A full overview of the functional annotation of all 11 shared loci is presented in Supplementary Table S3. The majority of the signals were regulatory (45 %), intronic (27 %) and intergenic (18 %). Three lead loci had supportive evidence from colocalization analysis for a shared causal genetic variant between ADRD and bilateral hippocampal volumes, including *TNIP1*, *SHARPIN* and *IGIP* (range posterior probability PP-H4 = 0.74–0.99, Supplementary Table S4). The lead variant located on an intron of *TNIP1* (TNFAIP3 Interacting Protein 1) (rs871269; conjFDR = 0.025) associated with gene expression of *LACTB*, *RAB8B* and *RPS27L* in multiple brain tissues (Supplementary Fig. S3). This potential protective variant for ADRD reached suggestive genome-wide significance in the Bellenguez study stage1 meta results (P = 3.7 × 10–5), and genome-wide significance in the stage1 and stage2 *meta*-analysis (P = 8.7 × 10–9) [15], demonstrating the potential for enhancing gene discovery power by integrating hippocampal volumes in this study.

**Fig. 3:**
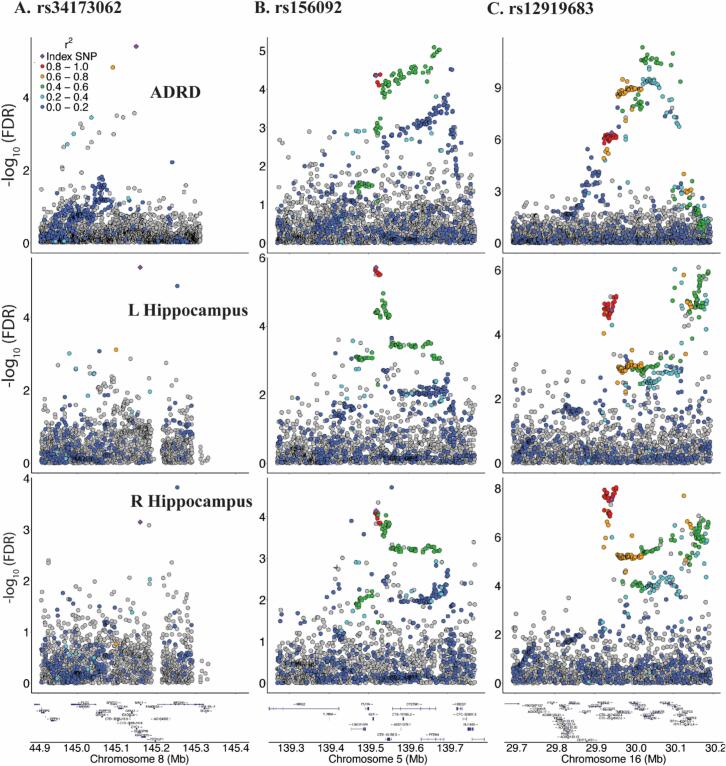
Regional locus plots: Single-nucleotide polymorphism-level associations with ADRD, bilateral hippocampal volumes at *SHARPIN*, *IGIP* and *KCTD13* locus, displayed as a locus zoom plot. The index SNP is annotated in purple, and nearby SNPs within 500 KB ranges are colored according to how strong they are in LD (r2) with the index SNP, as derived from the 1000 Genomes LD reference. A: Index SNP rs34173062; B: Index SNP rs156092; C: Index SNP rs12919683. ADRD: Alzheimer’s disease and related dementias; KB: Kilobyte; LD: linkage disequilibrium.

The lead variant, rs34173062 (conjFDR = 0.0245), is a missense variant located in an exon of *SHARPIN* and is a known AD risk locus involved in postsynaptic function (Fig. 3a) [26]. This variant acts as eQTL, with trans effects of rs34173062 (effect allele A) on increased gene *ZNF707* expression in the anterior cingulate. The lead variant rs156092 (conjFDR = 0.0215) is located on the previously reported AD risk gene *IGIP*, but this specific locus is new to AD. No QTL effects have been identified for this genetic variant. Another shared locus is rs12919683 (conjFDR = 0.00028), which is located on *KCTD13* (Fig. 3c). This locus seems to have multiple trans QTL effects, including effects on brain gene expression of *YPEL3*, *MAPK3*, *GDPD3*, *TBX6*, *INO80E*, *ASPHD1* and *BOLA2*, and sQTL effects *DOC2A* splicing in multiple brain tissues.

### 3.3 Functional interpretation based on Phenome‑wide association studies

**Fig. 4:**
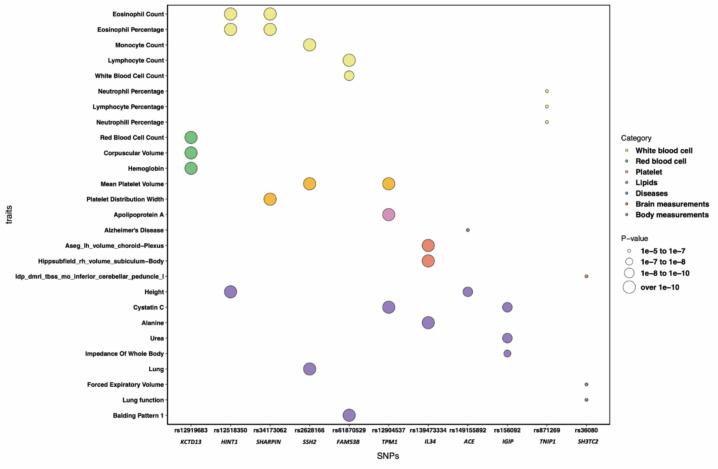
Functional interpretation based on Phenome‑wide association studies Top hits from the Phenome-wide association studies (PheWAS) were identified by searching for key pleiotropic traits associated with a list of SNPs with P < 1 × 10^–5^ in the MRC IEU OpenGWAS data. The x-axis indicates shared loci, while the y-axis represents related traits from the PheWAS analysis. The circle size corresponds to the p-values, and the colors represent different trait categories. The related traits belong to the following categories: white blood cell, red blood cell, platelet, lipids, diseases, brain measurements, and body measurements.

To identify phenotypic traits that have genetic pleiotropy with ADRD and bilateral hippocampal volumes, we performed phenome-wide association studies (PheWAS) for the 11 loci that were shared across ADRD and hippocampal volumes. By systematically analyzing all significant associations (P < 1 × 10–5) in the MRC IEU OpenGWAS database, we identified 442 associations for 11 shared AD-hippocampal volume loci. Most of the associations were found for *SSH2* (30.0 %), *KCTD13* (24.4 %), and *SHARPIN* (13.8 %) (Supplementary Table S5). Among all the associated traits, we found 228 (51.5 %) to be brain neuroimaging measurements. Further, 21.5 % of the PheWAS associations included measurements of the body (e.g., body composition, metabolomic, cardiovascular, and respiratory markers), followed by white blood cells (7.2 %), red blood cells (4.5 %), platelets (2.9 %) and lipids (2.7 %) (Supplementary Table S5). To identify key pleiotropic traits, we further examined the strongest associations for each locus (Fig. 4). Five loci, including *HINT1*, *SHARPIN*, *SSH2*, *FAM53B*, and *TNIP1*, showed the strongest associations with white blood cell markers, highlighting the role of immune-related processes in AD. Additionally, *KCTD13* showed the strongest associations with red blood cell markers.

### 3.4 Association evidence of lead shared loci across AD phenotypes

Of the 11 lead SNPs identified, three exhibited significant associations with AD–related biomarkers and neuropathology. The minor allele of rs871269, located within the *TINP1* locus, was positively correlated with CSF total Tau (β = 0.0225, P = 3.8 × 10^–2^) and phosphorylated Tau (β = 0.0348, P = 1.6 × 10^–3^) levels. At the *SHARPIN* locus, the variant rs34173062 demonstrated associations with CSF amyloid levels (β = 0.0527, P = 6.0 × 10^–3^) and Braak NFT stage (β = 0.1123, P = 6.2 × 10^–2^). Finally, rs149155892 in the *ACE* locus was inversely associated with CSF amyloid levels (β = –0.0984, P = 4.7 × 10^–3^) and positively associated with CERAD scores for neuritic plaque burden (β = 0.3708, P = 9.2 × 10^–4^). Detailed statistics for all 11 lead SNPs are provided in Table 2.

**Table 2:** Shared lead variants with results from AD CSF endophenotypes, AD related pathologies GWAS studies.

**Shared Loci**					**AD CSF endophenotypes (beta/p)**			**AD related pathologies (beta/p)**		
**Variant**	**Effect**	**Gene**	**Directions AD/Hippocampal volume**	**conjFDR**	**Amyloid**	**Tau**	**pTau**	**CERAD score for neuritic plaques**	**amyloid-β plaques**	**Braak NFT stage**
rs12518350	A	*HINT1*	-/+	0.0329	0.0057/6.1e-01	−0.0049/6.7e-01	−0.0112/3.3e-01	−0.0283/4.6e-01	0.005/9e-01	−0.0454/2.0e-01
rs156092	T	*IGIP*	-/-	0.0215	0.006/5.7e-01	2e-04/9.8e-01	−0.0053/6.2e-01	0.0173/6.2e-01	0.0429/2.5e-01	0.0094/7.7e-01
rs36080	A	*SH3TC2*	-/+	0.0351	0.0149/2.1e-01	−0.0014/9.1e-01	−0.0043/7.2e-01	0.0387/3.3e-01	0.0382/3.7e-01	−0.0154/6.7e-01
rs871269	T	*TNIP1*	/+	0.0245	−0.0146/1.8e-01	**0.0225/3.8e-02**	**0.0348/1.6e-03**	−0.0012/9.7e-01	−0.0197/6.1e-01	−0.0381/2.5e-01
rs34173062	A	*SHARPIN*	+/-	0.00216	**0.0527/6.0e-03**	−0.0122/5.2e-01	−0.0311/1.0e-01	−0.0024/9.7e-01	0.0037/9.5e-01	**0.1123/6.2e-02**
rs61870529	T	*FAM53B*	+/-	0.0105	0.0092/4.3e-01	− 0.0032/7.8e-01	− 0.001/9.3e-01	0.0346/3.6e-01	0.0307/4.6e-01	0.0165/6.5e-01
rs12904537	T	*TPM1*	+/-	0.00977	−0.0069/5.1e-01	−0.0038/7.1e-01	−0.0028/7.9e-01	−0.005/8.9e-01	0.0472/2.4e-01	0.0283/3.9e-01
rs12919683	T	*KCTD13*	+/+	0.00028	−0.0136/2.4e-01	−0.0113/3.3e-01	−0.013/2.7e-01	−0.0072/8.3e-01	0.0037/9.1e-01	0.0134/6.7e-01
rs139473334	A	*IL34*	-/-	0.00283	NA	NA	NA	−0.0211/5.5e-01	−0.0486/2.1e-01	0.0449/1.7e-01
rs149155892	T	*ACE*	+/-	0.0162	**−0.0984/4.7e-03**	0.0124/7.2e-01	0.0394/2.6e-01	**0.3708/9.2e-04**	0.0977/4.1e-01	0.127/2.2e-01
rs2628166	T	*SSH2*	+/+	0.0439	−0.0097/3.3e-01	0.011/2.7e-01	0.0135/1.8e-01	−0.0031/9.2e-01	0.0106/7.7e-01	0.0125/6.9e-01

Numbers highlighted in bold show significance in the studies compared; significance level defined as p < 0.1 for GWAS study AD CSF endophenotypes and related pathologies due to the limited sample size in both GWAS analysis. Direction of effect has been harmonized across all summary statistics in reference to listed effect allele. 1. AD CSF endophenotypes: GWAS of multiple neuropathology endophenotypes identifies new risk loci and provides insights into the genetic risk of dementia [24]. 2. AD related pathologies: Shade et al. [25].

## 4 Discussion

In this study, we aimed to uncover the shared genetic architecture between ADRD and bilateral hippocampal volumes. By leveraging the pleiotropic enrichment of these two traits, we were able to boost power and identify 11 loci jointly associated with ADRD and hippocampal volume. Notably, our findings included 9 novel candidate risk loci for ADRD, including *KCTD13*, *IGIP*, *HINT1*, *SH3TC2*, *FAM53B*, *TPM1*, *IL34*, *ACE* and *SSH2*. In addition to these novel findings, we replicated two previously established AD loci—*TNIP1* and *SHARPIN* [15,27]Our findings advance the catalog of ADRD-associated genetic variants and provide insights into the biological overlap between hippocampal volume and dementia risk, thereby supporting the use of imaging endophenotypes in genetic studies of neurodegenerative disease.

We identified two known ADRD loci to be associated with hippocampal volume, one of them including *TNIP1* (rs871269) located on chromosome 5 [15]. *TNIP1* has been associated with hippocampal sclerosis and amyotrophic lateral sclerosis (ALS) [28]. Its product, *TNIP1*, has an inflammatory function, but it has also been expressed in hippocampal and cerebellar areas during the development of the central nervous system, highlighting its crucial role in the development of brain[29]. Previous studies have indicated that the causal genetic variant for AD and ALS underlying *TNIP1* may be the nearby-located gene *GPX3* [30]. Accordingly, one recent study reported that *TNIP1* (rs34294852) affects levels of CSF GPX3, a protein that protects against oxidative stress, as AD progresses. Here, we report that *TNIP1* also may influence gene expression of *LACTB*, *RAB8B* and *RPS27L*, of which the latter two are both involved in autophagy[31,32]. A recent community study found that the cortical protein, *LACTB* is implicated in AD [33]. Moreover, we now show that the minor allele of rs871269 on *TNIP1* is significantly correlated with higher CSF total and phosphorylated Tau levels. Further research is needed to understand through which specific immune-related mechanisms *TNIP1* influences hippocampal volume and risk for AD.

The second known ADRD locus, rs34173062 on *SHARPIN*, has been associated with late-onset AD in various ethnic groups [34]. This missense variant is involved in postsynaptic function[26]. Another GWAS study showed that the medial temporal circuit could be used as another imaging phenotype and the SNP rs34173062 on the *SHARPIN* gene had a genetic modifying effect on its atrophy [35]. Those shared variants may play a role in AD pathogenesis by affecting hippocampal volumes, which is instrumental in refining their interrelationships. Here, we extend these findings to molecular biomarkers, demonstrating that rs34173062′s minor allele associates with increased CSF amyloid levels and a trend toward higher Braak neurofibrillary tangle stage, linking *SHARPIN* variation not only to structure but also to hallmark AD pathology.

Additionally, we have identified 9 potential novel ADRD loci. One intriguing shared signal was *KCTD13*, which is located on the 16p11.2 locus. *KCTD13* belongs to the KCTD family, of which many members are associated with neuropsychiatric disorders [36]. *KCTD13* has been documented to act as a cullin-3 adaptor for the ubiquitination and degradation of RhoA, a crucial small GTPase protein regulating the actin cytoskeleton, pivotal for neuronal development and synaptic function. Deletion of the entire *KCTD13* gene results in elevated RhoA expression, loss of dendritic spines, and diminished synaptic activity in hippocampus CA1 region [37]. RNA-seq analyses of gene expression profiles from the cortex and hippocampus of KCTD13-deficient (exon 2-deleted) mice unveiled altered signaling pathways critical for neurodevelopment, including synaptic formation and reduced spine density in the hippocampus[37,38]. Altogether, this suggests that *KCTD13* may impact risk for AD risk by influencing neuronal development and synaptic function of the hippocampus.

Of the 11 genetic variants that were shared between ADRD and hippocampal volume, 7 signals had discordant effect directions, indicating that the minor allele was associated with increased risk for ADRD and smaller hippocampal volumes (and vice versa, where decreased risk for ADRD associates with larger hippocampal volumes). This association between smaller hippocampal volumes at middle-age and increased risk for AD is in line with brain capacity theory[39,40]. According to this theory, grey matter volume is a potential quantitative brain reserve, indicating that larger brain volumes protect against the consequences of neuronal degeneration[41]. The 4 loci with concordant effects (i.e., increased ADRD risk and larger hippocampal volumes) imply more complicated neurodevelopmental process in the life stage. For instance, larger hippocampal volumes have been found to correlate with autism and children with fragile X syndrome[42], and these neurodevelopmental conditions are believed to share genetic and mechanistic overlaps with AD[43].

Our analysis included GWAS summary statistics for both left and right hippocampal volume, separately. While most results were consistent across the left and right hippocampal hemispheres, three loci were specific to left hippocampal volumes (*HINT1*, *TPM1* and *SSH2*), and one to right hippocampal volume (*SH3TC2*). Subcortical volume asymmetry seems an important aspect of brain organization and multiple genetic variants have been identified to be associated with asymmetry of the brain[44]. Furthermore, subcortical volume asymmetry plays an important role in AD, as asymmetry increases as the disease progresses[43]and this links to specific cognitive deficits[45].

Our study has certain limitations. Firstly, there may be overlap in the samples used across the GWAS in our cross-trait analysis, which could lead to overestimating the effects of shared signals. While we used the largest GWAS summary statistics available for our current analyses, independent data is needed to validate identified shared loci in the future. Second, while our research provides genetic evidence for a shared role of immune cell and lipids markers in ADRD and hippocampal volumes, the specific pathway involved in this process needs experimental validation.

In summary, our study integrated GWAS summary statistics on ADRD and bilateral hippocampal volumes to uncover the shared genetic architecture between these traits. We identified two known AD loci located in *SHARPIN* and *TNIP1*, along with nine novel risk loci, including *KCTD13* as shared genetic variants. Through gene annotation and QTL analysis, we pinpointed the potential mapped genes for these shared loci, most of which are protein-coding. Additionally, we highlighted the significant roles of immune cells and lipid markers as shared genetic mechanisms in the hippocampus-ADRD pathway.

## 5 Conclusion

In this study, we identified known ADRD loci located in *SHARPIN* and *TNIP1*, along with nine novel risk loci − including *KCTD13* – that are shared between Alzheimer’s disease and hippocampal volume. Through gene annotation and eQTL analysis, we mapped these to potential target genes, most of which are protein-coding. Moreover, we highlighted the significant involvement of immune cells and lipid markers, supporting shared biological mechanisms in the hippocampus-ADRD pathway. Reported findings advance our understanding of the genetic architecture of ADRD, which aids in the identification of potential targets for functional follow-up studies and future therapeutic development. Furthermore, they may help refine polygenic risk score models, thereby strengthening the translational relevance of our study for early detection and treatment strategies in Alzheimer's disease and related dementias.

## CRediT authorship contribution statement

**Chenyang Jiang:** Writing – review & editing, Writing – original draft, Visualization, Methodology, Investigation, Conceptualization. **Sven J. van der Lee:** Writing – review & editing, Methodology. **Niccolò Tesi:** Writing – review & editing, Methodology. **Wiesje M. van der Flier:** Writing – review & editing. **Betty M. Tijms:** Writing – review & editing, Methodology. **Lianne M. Reus:** Writing – review & editing, Supervision, Methodology.

## Declaration of competing interest

W.F. has been an invited speaker at Biogen MA Inc, Danone, Eisai, WebMD Neurology (Medscape), NovoNordisk, Springer Healthcare, European Brain Council. All funding is paid to her institution. W.F. is consultant to Oxford Health Policy Forum CIC, Roche, Biogen MA Inc, and Eisai. All funding is paid to her institution. W.F. participated in advisory boards of Biogen MA Inc, Roche, and Eli Lilly. W.F. is member of the steering committee of EVOKE/EVOKE+ (NovoNordisk). All funding is paid to her institution. W.F. is member of the steering committee of PAVE, and Think Brain Health. W.F. was associate editor of Alzheimer, Research & Therapy in 2020/2021. W.F. is associate editor at Brain. All other authors report no biomedical financial interests or potential conflicts of interest.
